# Supplementary figures and images for: A small step to discover candidate biological control agents from preexisting bioresources by using novel nonribosomal peptide synthetases hidden in activated sludge metagenomes
Source: PLoS One. 2023 Nov 27;18(11):e0294843. doi: 10.1371/journal.pone.0294843 (PMC10681181; doi:10.1371/journal.pone.0294843)

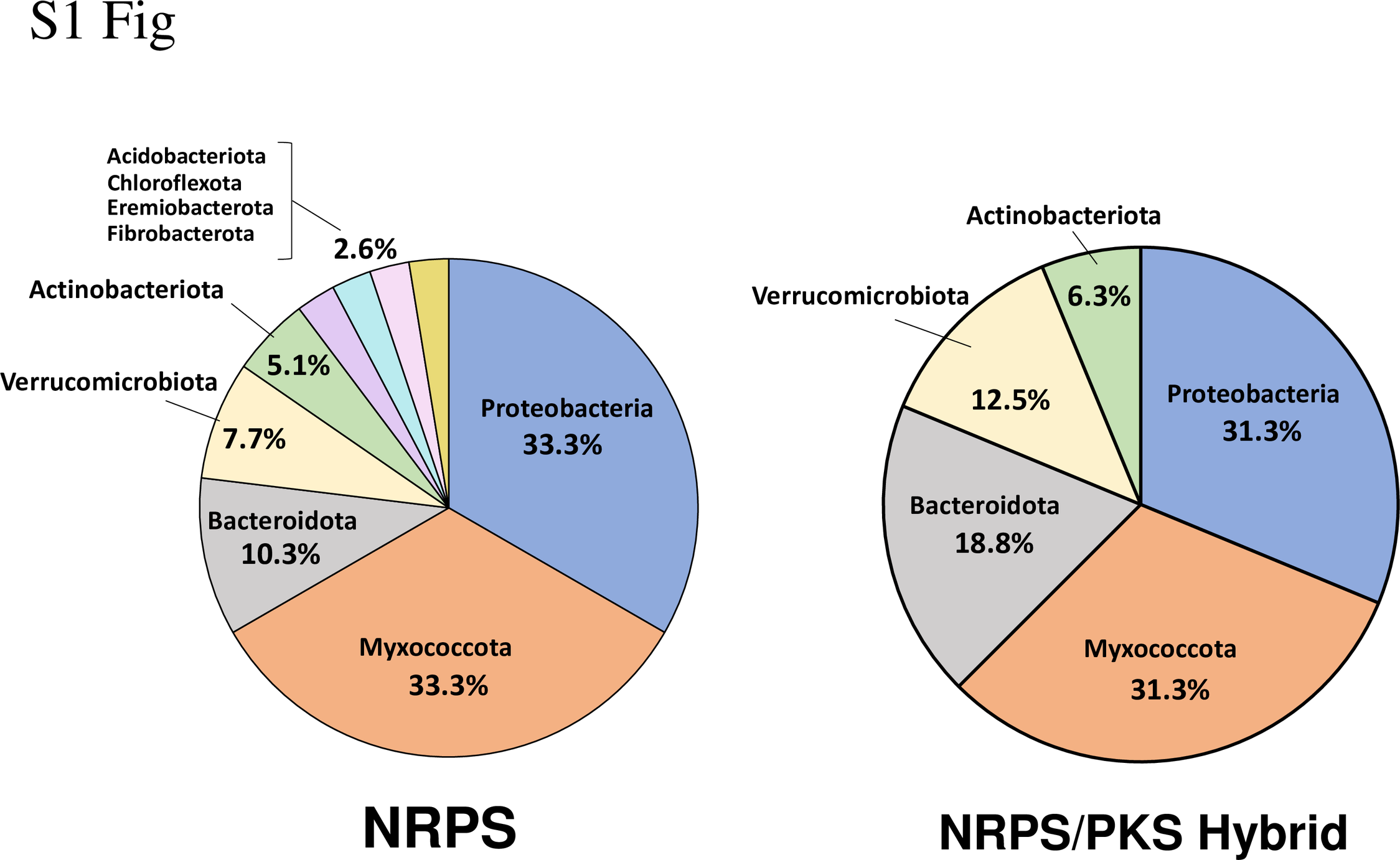

Supplement: S1 Fig — (TIF) [file pone.0294843.s004.tif]

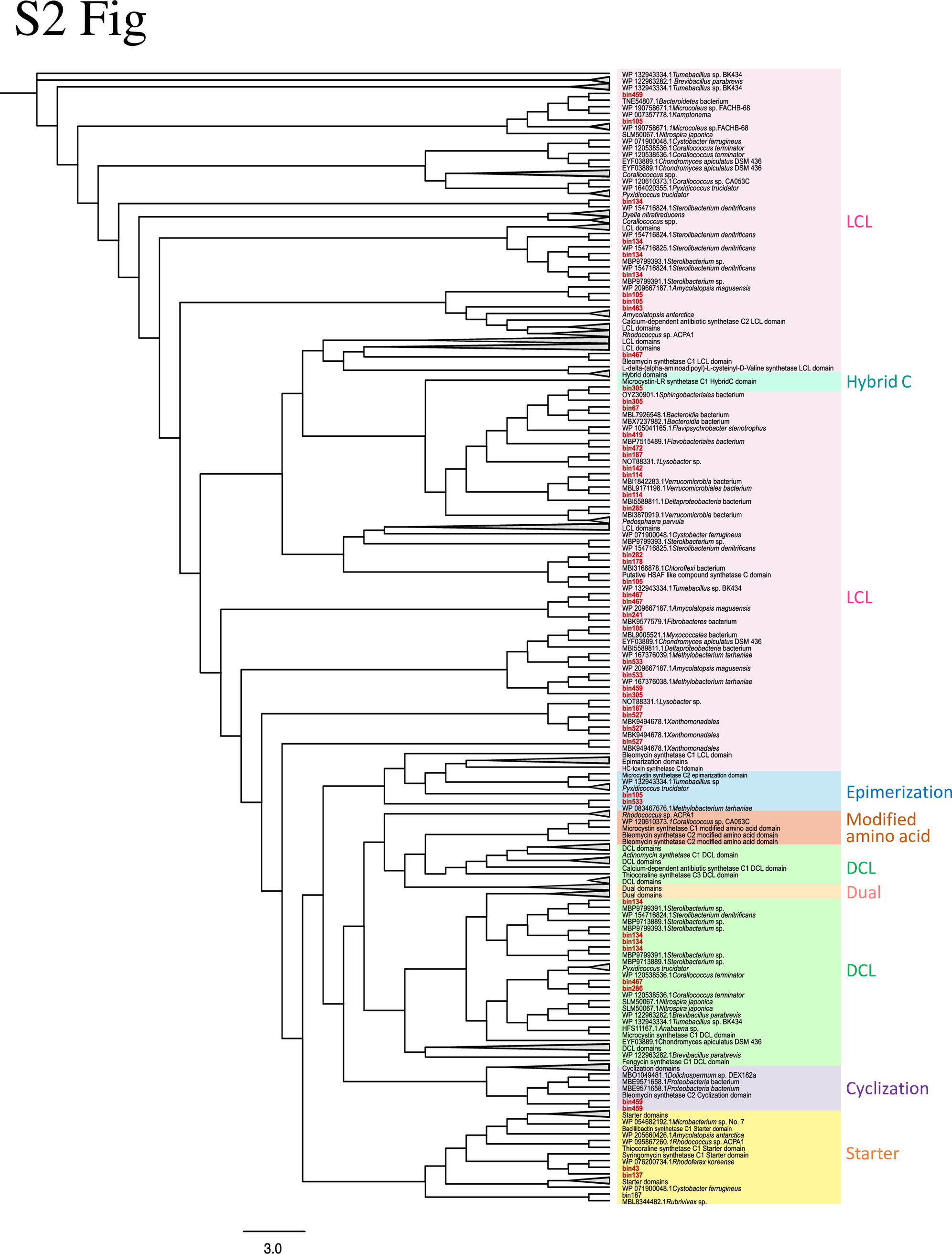

Supplement: S2 Fig — (TIF) [file pone.0294843.s005.tif]

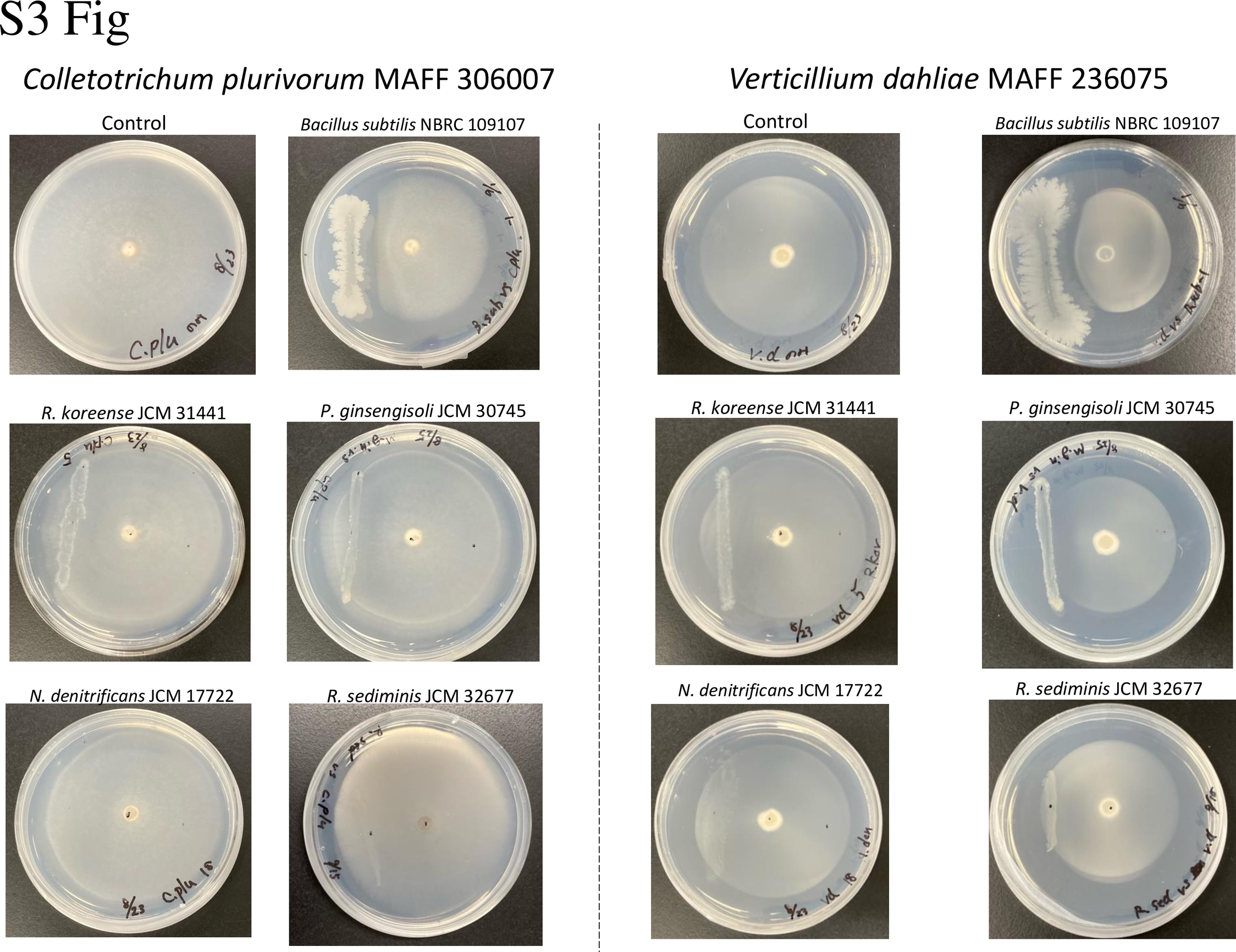

Supplement: S3 Fig — An overnight culture of each bacterial strain was inoculated in a straight line on an R2A agar plate with an inoculating loop. (TIF) [file pone.0294843.s006.tif]

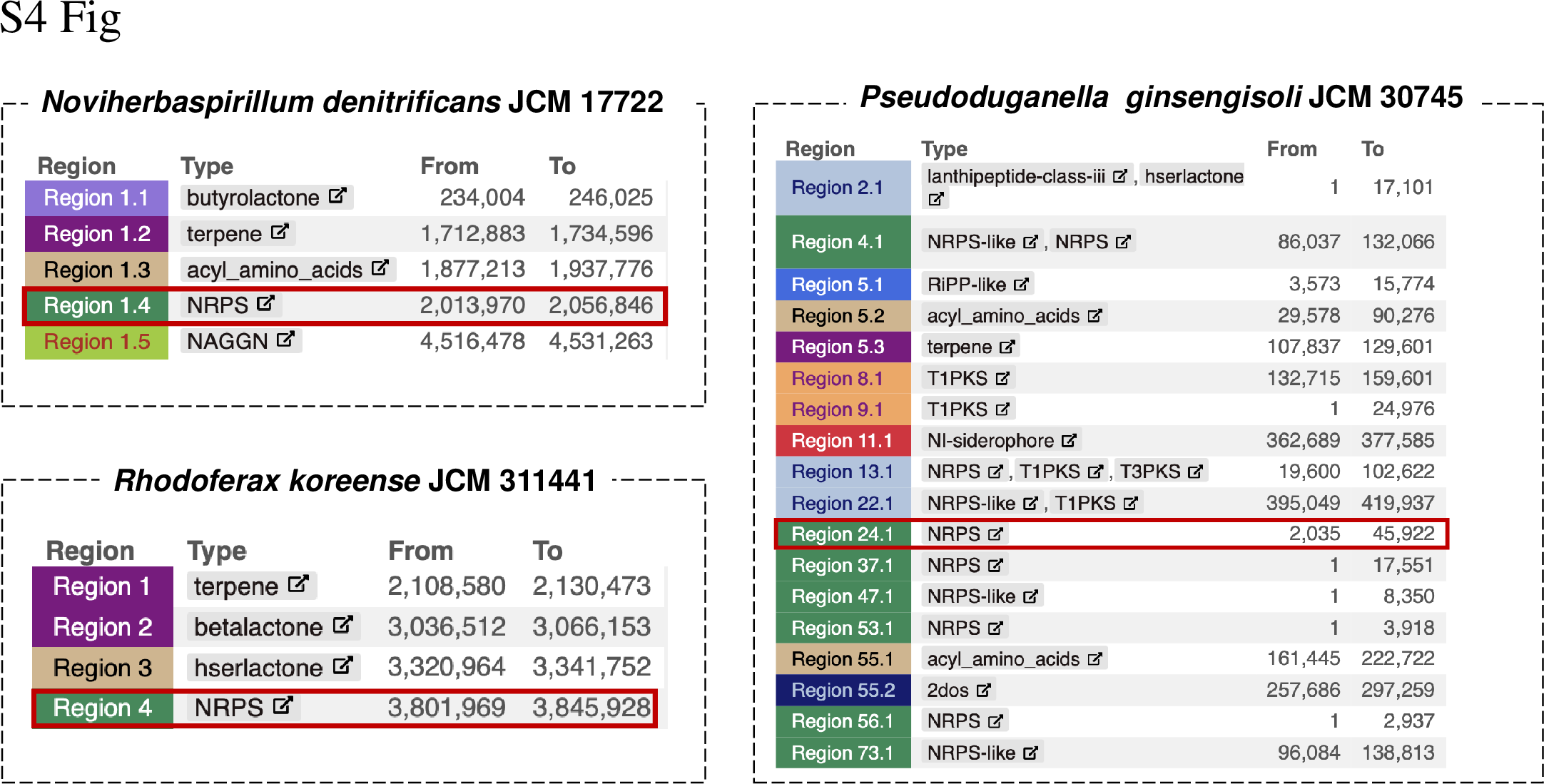

Supplement: S4 Fig — The red box shows NRPS genes with the C domain of syringomycin synthetase. (TIF) [file pone.0294843.s007.tif]
