# Supplementary material for: A small step to discover candidate biological control agents from preexisting bioresources by using novel nonribosomal peptide synthetases hidden in activated sludge metagenomes
Source: PLoS One. 2023 Nov 27;18(11):e0294843. doi: 10.1371/journal.pone.0294843 (PMC10681181; doi:10.1371/journal.pone.0294843)

New strategy for discovering candidates for biological control agents with NRPS

Metagenomic shotgun sequencing, assembly, and binning

Screening NRPS and NRPS/PKS hybrid gene cluster

Selecting the cultured microbial resources with NRPS

Evaluation of actual antifungal activity

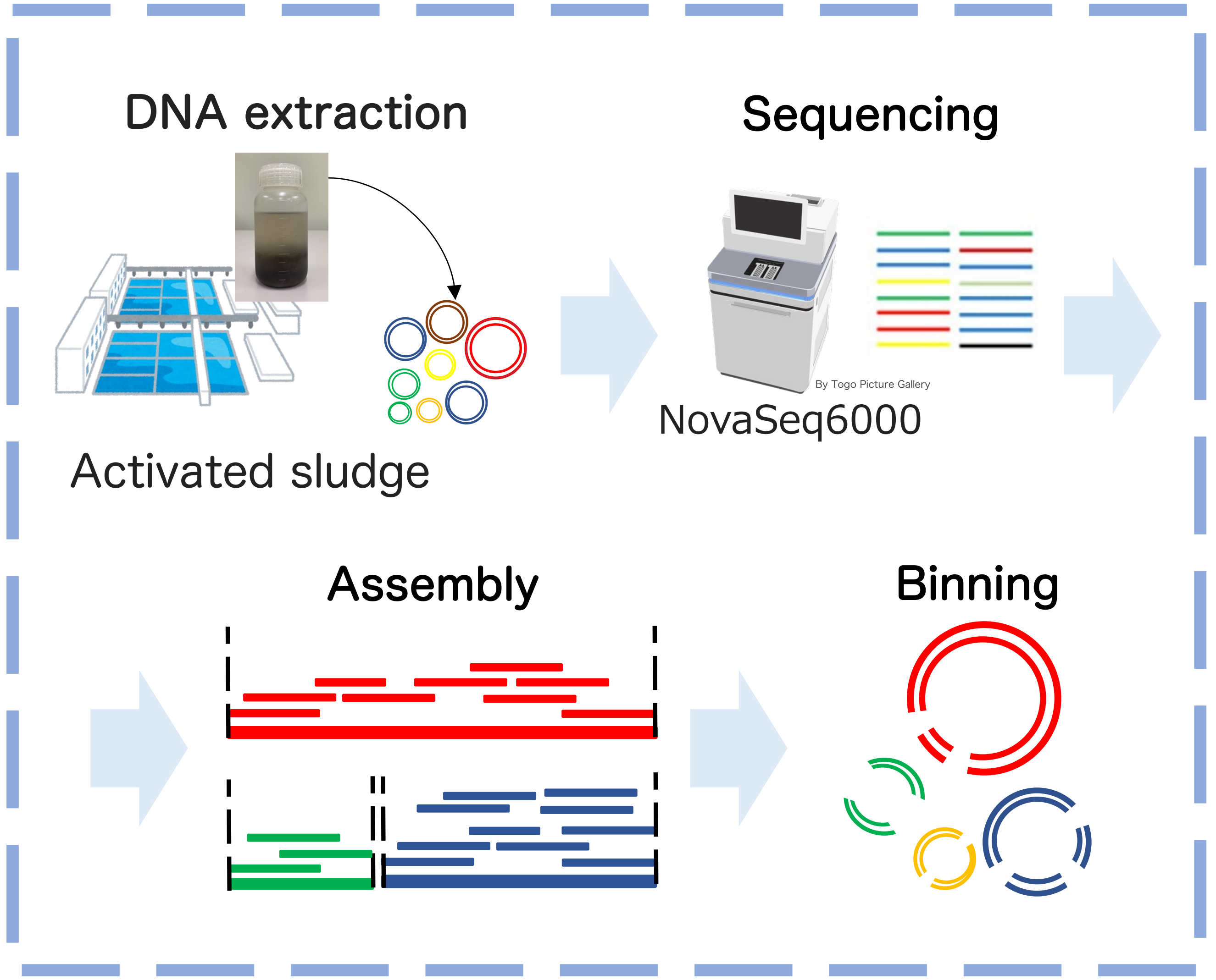

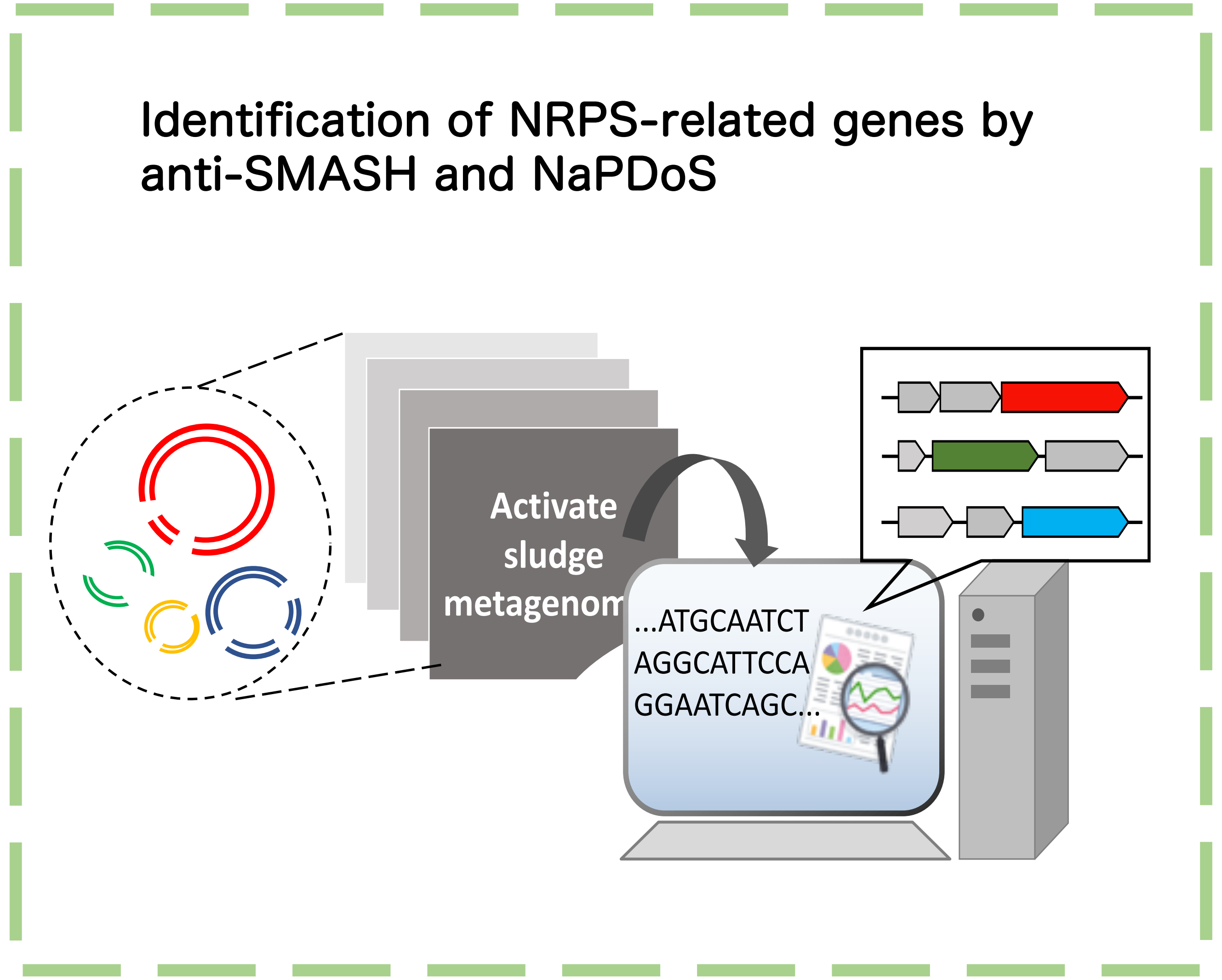

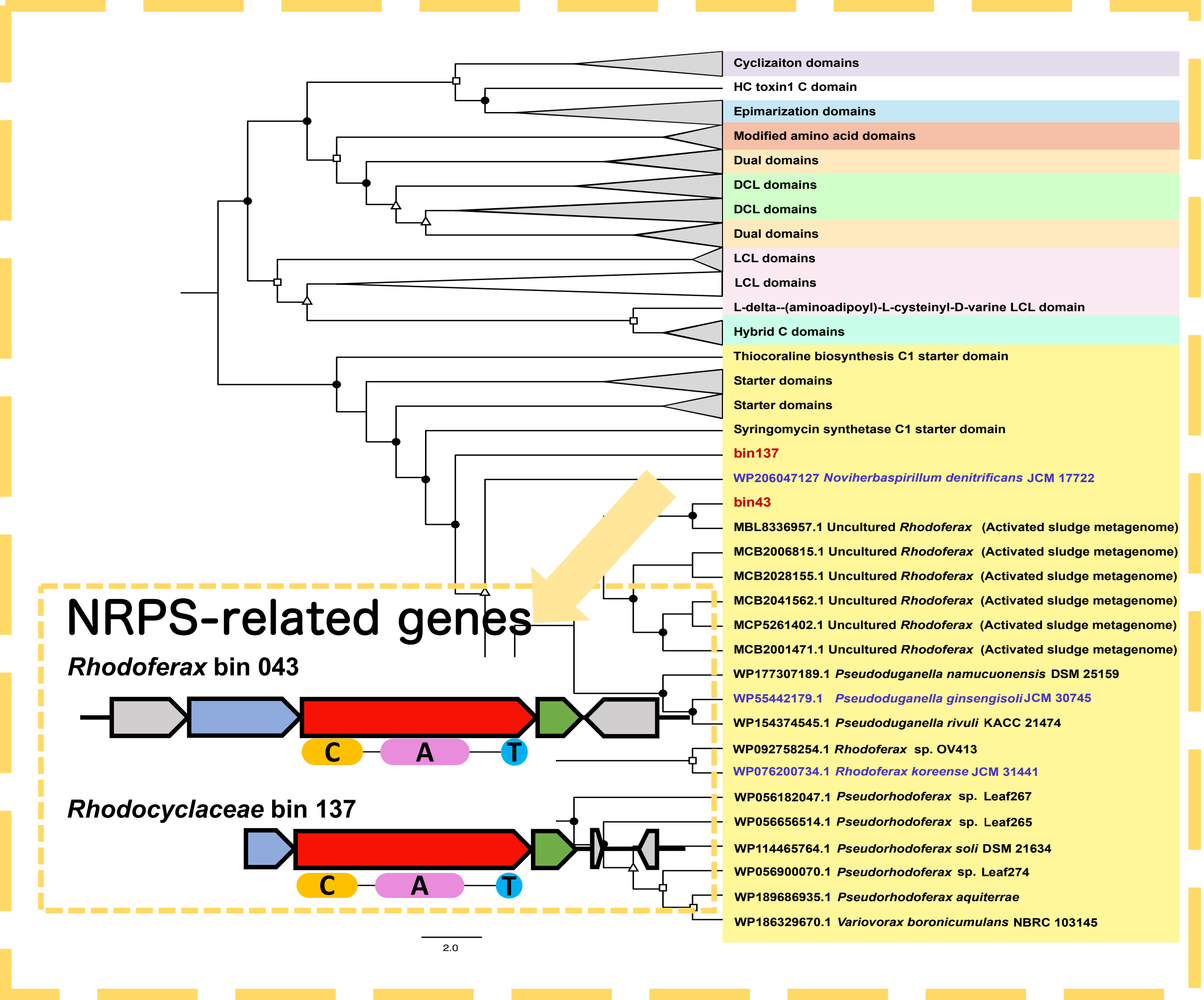

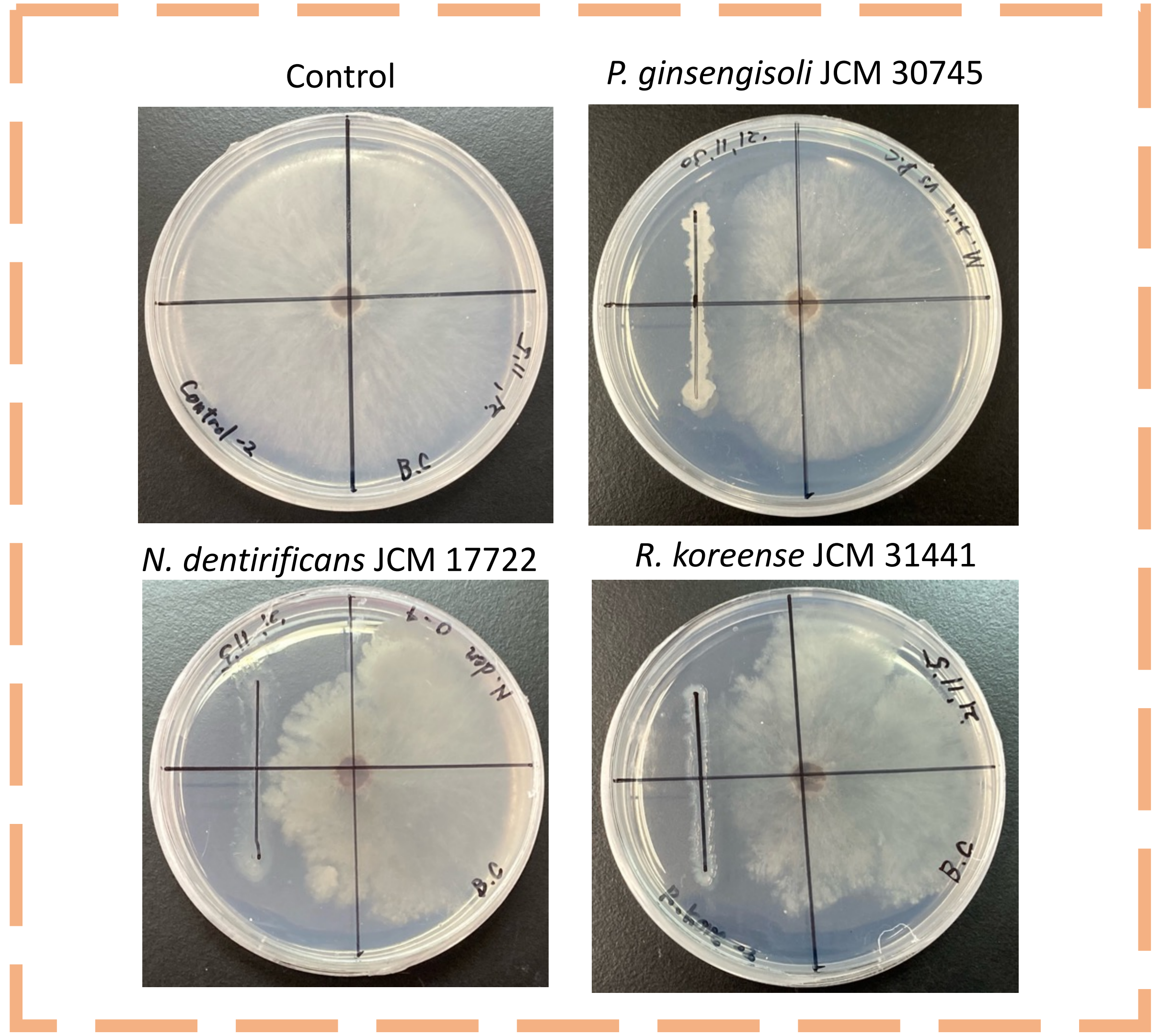

Supplement: S1 Graphical abstract — (PDF) [file pone.0294843.s008.pdf]
