# Supplementary material for: A small step to discover candidate biological control agents from preexisting bioresources by using novel nonribosomal peptide synthetases hidden in activated sludge metagenomes
Source: PLoS One. 2023 Nov 27;18(11):e0294843. doi: 10.1371/journal.pone.0294843 (PMC10681181; doi:10.1371/journal.pone.0294843)
Supplement: Attachment — Submitted filename: 2023-06-12 Manuscript Number_ PONE-D-23-16870.pdf [file pone.0294843.s009.pdf]

Manuscript Number: PONE-D-23-16870
Title: A small step to discover candidate biological control agents from pre-existing bioresources by using novel nonribosomal peptide synthases hidden in the activated sludge metagenomes

The authors described a small-scale test case to select NRPS or NRPS/PKS clusters from activated sludge shotgun metagenome data. Next, they looked at culture collections for strains that harbor the selected clusters, with the end goal to use these strains as biological control agents. Next, they investigated the antifungal activity of these strains. Even though the authors identified three strains that harbor a similar NRPS gene between them, the claim that the product of this NRPS is responsible for the antifungal activity is weak, and further experiments are needed to corroborate with this hypothesis. Moreover, some sections must be re-written to be more clear about how and why the analysis was performed.

General points:

Abstract: the abstract should be re-written. E.g. (**Pg. 8, Ln. 22-26**), it is unclear the connection between the culture collection strains and the shotgun metagenome data; (**Pg. 9, Ln. 37-41**), the conclusion is too vague. The problem persists through the introduction topics.

Pg. 14-15, Ln. 135-152/ Pg.19, Ln. 223-231/ Pg. 22, Ln. 260-263: section on C-domain phylogeny approach should be re-written or removed. No information is obtained from this analysis.

Pg. 17, Ln. 197-198: it would be interesting to discuss the NRPS and NRPS/PKS domain architecture in this section. E.g., do they have a complete set of modules, how many?

Pg. 23, Ln. 285-300: even though there might be a correlation between the presence of this NRPS with the antifungal activity, the results presented in this section are not enough to claim it. There are many options to follow: 1. perform reverse genetics in one of the bacterial strains and delete the NRPS, and then repeat the antifungal assay. 2. grow at least one of the strains or perform heterologous expression to isolate the NRPS product, then repeat the antifungal assay it the purified molecule. If none of these are done, this section should be toned down.

Pg. 24, Ln. 305-313: it would be more clear if the annotation of genes belonging to the NRPS gene cluster to be discussed before, in the first section of the results.

Specific points:

Title: correct nonribosomal peptide *synthase* to nonribosomal peptide *synthetase*.

Pg. 18, Ln. 203: correct *181* to *180*.

Pg. 18, Ln. 215-217: phylum names should not be italicized. Problem persists throughout the whole manuscript.

Pg. 18, Ln. 217-218: what does it mean *NRPS and NRPS/PKS hybrid gene clusters, with each 31-33% proportion*? Sentences must be re-written.

Pg. 19, Ln. 222: replace *unused* by *underexploited*.

Pg. 21, Ln. 237: replace *Myxococcota are* by Myxococcota is.

Pg. 21, Ln. 253: replace *including* by *producing*.

Pg. 22, Ln. 269-270: genera names should not be abbreviated here.

Pg. 23, Ln. 297: SI figure says *Massilia ginsengisoli*. Please check the correct genera name.
