# Supplementary material for: A small step to discover candidate biological control agents from preexisting bioresources by using novel nonribosomal peptide synthetases hidden in activated sludge metagenomes
Source: PLoS One. 2023 Nov 27;18(11):e0294843. doi: 10.1371/journal.pone.0294843 (PMC10681181; doi:10.1371/journal.pone.0294843)
Supplement: Attachment — Submitted filename: Response to Reviewers-R2.pdf [file pone.0294843.s011.pdf]

PONE-D-23-16870R1

A small step to discover candidate biological control agents from preexisting bioresources by using novel nonribosomal peptide synthetases hidden in the activated sludge metagenomes

Shun Tomita[1*], Kyohei Kuroda[1], Takashi Narihiro[1*]

1 Bioproduction Research Institute, National Institute of Advanced Industrial Science and Technology (AIST), Toyohira-ku, Sapporo, Hokkaido, Japan

＊Co-corresponding authors e-mail addresses: tomita.s@aist.go.jp (ST), t.narihiro@aist.go.jp (TN).

Response to the Journal requirements:

Please review your reference list to ensure that it is complete and correct. If you have cited papers that have been retracted, please include the rationale for doing so in the manuscript text, or remove these references and replace them with relevant current references. Any changes to the reference list should be mentioned in the rebuttal letter that accompanies your revised manuscript. If you need to cite a retracted article, indicate the article's retracted status in the References list and also include a citation and full reference for the retraction notice.

Response:

We have reviewed and modified the reference list under the PLOS ONE's style requirements (p23:L408, 413, p24:L438, p25:L443, p26:L464, 467, p29:L529, p30:L543, 547, 551, p31:L559, p32:L584).

Response to the Editor and Reviewers

We sincerely appreciate the valuable and positive comments on our revised manuscript from the Editor and Reviewers. A point-by-point response to the comments is given below. And we have added the Figure 4 caption, which did not explain negative control. In addition, as you can see in the attached certificate, we have Elsevier Language Editing Service proofread the manuscript. Please note that the Line numbers in the responses correspond to the revised manuscript with changes marked.

Reviewer comments:

1.My previous points were addressed, but unfortunately the manuscript was not written with due care. Strain names were repeatedly misspelled and abbreviated incorrectly (Noviherbaspirillum denitrificans is correct, not dentrificans or denitirificans, B. subtilis not Ba. subtilis, etc.), even in the revised version. I do not consider it my responsibility as a reviewer to correct all these errors, but I strongly recommend that they be corrected before the manuscript is published. The authors should consider having a native speaker proofread the manuscript (in the best case, this should happen before the first submission).

Response

We apologize for the mistakes. We have modified the strain names (Fig 3,4, S3 Fig, and Throughout the manuscript). In addition, the manuscript has been edited by an Elsevier Language Editing Service, as you can see in the attached certificate.

2.Figure 4: The authors should mention that *R. sediminis* JCM 32677 was used as a negative control in the figure caption.

Response

We apologize for lack of the explanation of negative control. We have added the Figure 4 caption (p21: L370-371).
